# Instrument design and protocol for the study of light controlled processes in aquatic organisms, and its application to examine the effect of infrared light on zebrafish

**DOI:** 10.1371/journal.pone.0172038

**Published:** 2017-02-17

**Authors:** Marcus P. S. Dekens, Nicholas S. Foulkes, Kristin Tessmar-Raible

**Affiliations:** 1 Max Perutz Laboratories, Vienna Biocenter, University of Vienna, Vienna, Austria; 2 Karlsruhe Institute of Technology, Eggenstein, Germany; 3 Centre for Organismal Studies, University of Heidelberg, Heidelberg, Germany; 4 Research Platform “Rhythms of Life”, University of Vienna, Vienna, Austria; University of Lübeck, GERMANY

## Abstract

The acquisition of reliable data strongly depends on experimental design. When studying the effects of light on processes such as behaviour and physiology it is crucial to maintain all environmental conditions constant apart from the one under study. Furthermore, the precise values of the environmental factors applied during the experiment should be known. Although seemingly obvious, these conditions are often not met when the effects of light are being studied. Here, we document and discuss the wavelengths and light intensities of natural and artificial light sources. We present standardised experimental protocols together with building plans of a custom made instrument designed to accurately control light and temperature for experiments using fresh water or marine species. Infrared light is commonly used for recording behaviour and in electrophysiological experiments although the properties of fish photoreceptors potentially allow detection into the far red. As an example of our experimental procedure we have applied our protocol and instrument to specifically test the impact of infrared light (840 nm) on the zebrafish circadian clock, which controls many aspects of behaviour, physiology and metabolism. We demonstrate that infrared light does not influence the zebrafish circadian clock. Our results help to provide a solid framework for the future study of light dependent processes in aquatic organisms.

## Introduction

Light affects most life on earth. However, many of the functions and mechanisms underlying these effects are far from understood. The vast extent of adaptations can be predicted from the widespread occurrence of light receptors with unknown functions in taxa spanning large evolutionary distances [1–5]. The advent of new genome editing technologies guarantees the development of a range of new model organisms for the study of light controlled processes. Furthermore, the broad scope of available aquatic species opens many possibilities for comparative studies addressing specific light controlled functions. Thus, research can be performed on species that inhabit very different light-environments, for example lunar rhythms are studied in a marine annelid [6], and the functions of photoreceptors have been studied in cavefish [7]. Furthermore, the two teleost species zebrafish and medaka are well-established genetic models for studying the effects of light. Zebrafish embryos already become light sensitive at 5 hours post fertilisation (h.p.f.), far before the onset of retinal development [8, 9]. Zebrafish cells, tissues and organs are directly light responsive [10], but the photoreceptors underlying potentially a wide range of light-dependent functions have so far not been functionally characterized. The diurnal zebrafish and medaka have also been established as useful models for studying circadian rhythms and the circadian clock, which first emerge during their early development [11, 12]. The environmental cues that set these clocks and thereby synchronise them with the environment are called “zeitgebers”. Light in most organisms including zebrafish plays the role of the principal zeitgeber [13].

Understanding the link between molecular mechanisms, brain circuits, and behaviour is an important aim for many photo- and chronobiologists. Rhythmic locomotor activity is therefore often assayed as a circadian clock output, typically recorded under infrared (IR) light. Although blue light receptors have been implicated in the entrainment of the circadian clock [14], it has been shown that rhythmic circadian clock gene expression can also be set by visible red light [15, 16]. In addition, some lampreys, fishes, amphibians and reptiles have been shown to contain a chromophore that predicts an ability to detect in the far red [17, 18], and thus IR light might potentially act as a zeitgeber. However, to our knowledge there is no published record on the absence or presence of the effects of IR light on the teleost circadian clock. The only references about the effects of IR light on zebrafish behaviour were published in the context of the visual system and at a time when the broad light sensitivity of its tissues and the widespread occurance of non-visual photoreceptors were unknown [19, 20].

The high sensitivity of aquatic ectotherms for light [10, 21] and temperature [12, 22, 23] makes them particularly susceptible to small fluctuations in these environmental conditions. Thus a technical resource and standardised method that minimizes undesired experimental variables is essential for addressing the various questions of photo- and chronobiological research. To meet this requirement we have designed, tested and optimized an experimental setup for reliably studying the influence of light on the photo- and chronobiology of aquatic animals. Commercial systems (e.g. from Noldus Information Technology) exist, but are highly expensive and thus not easily affordable for all labs that would benefit from the usage of the system. In addition, these systems have been solely designed for behavioural studies, and thus are not a multi-purpose tool that can be easily applied for sampling in order to investigate transcriptomic or proteomic changes in parallel to behaviour. Here we present a set of construction drawings and detailed descriptions for an instrument that controls environmental conditions together with an evaluation of how to apply and measure light. With these instructions a standard laboratory workshop can build the instrument, and it will cost about ten times less than current commercial solutions. As behavioural studies using IR light have become more common in aquatic species we applied our instrument and standardised protocol to determine the effect of IR light (840 nm) on the zebrafish circadian clock while at the same time demonstrating the features of our instrument. The instrument and data will be useful for a broad scientific community, since photo- and chronobiology are relevant to many research fields extending from cell proliferation, physiology and behaviour to immunology, metabolism, toxicology and chronopharmaceutics.

## Methods

### Zebrafish husbandry

Animal care was conducted according to Austrian and European guidelines for animal research (fish maintenance and care approved under: BMWFW-66.006/0012-WF/II/3b/2014). Work with zebrafish larvae under different light conditions is further approved by the experimental plan BMWFW-66.006/0003-WF/V/3b/2016, which is cross-checked by: Geschäftsstelle der Kommission für Tierversuchsangelegenheiten gemäß § 36 TVG 2012 p. A. Veterinärmedizinische Universität Wien, A-1210 Wien, Veterinärplatz 1, Austria, before being issued by the BMWFW. Zebrafish (*Danio rerio*) wild type strains were kept in a constant recirculating system at approximately 28°C [24]. Zebrafish were bred using standard protocols [25].

### Experimental conditions

Zebrafish embryos were placed in 50 ml cell-culture flasks (Greiner bio-one, Cellstar, red standard cap without filter) in a total volume of 20 ml E3 water [25]. For each condition one Greiner flask was used per biological replicate and three biological replicates were prepared for each zeitgeber time (ZT) analyzed. Six embryos were placed in each flask. Control samples that should remain in darkness were wrapped in a double layer of aluminium foil and placed in the same tank with the IR light treated samples. The samples were transferred to the temperature-controlled tank (28.0 ± 0.2°C) in the instrument well before 5 h.p.f because at that stage zebrafish embryos become light responsive [9]. To monitor the temperature in the setup during the course of the experiment the HOBO pendant light and temperature data logger (UA-002-64) was submerged next to the samples in the water. To obtain a reliable indication of the gene expression levels over the day the larvae were exposed to 12:12 hour infrared light-dark (IRD) or white light-dark (LD) cycles, and controls were kept in constant darkness (DD). Larvae from all conditions were sampled on day 6 at evenly distributed zeitgeber times; ZT3, ZT9, ZT15, and ZT21. To collect the larvae, the contents of the Greiner flask were poured over a mesh and transfered to a 2 ml reaction tube. Nearly all the water was removed and the sample was directly frozen in liquid nitrogen. The samples were stored at -80°C until all samples were collected in order to process them together and prevent variations due to differences in processing.

### Light measurement

IR and white light LEDs were installed as described. The spectrum and light intensity of the light was measured with the ILT950 spectrometer from International Light Technologies Inc. (Peabody, U.S.A.). We measured the light intensity at the position of the sample as the intensity depends on the distance from the source. The light intensity is given in irradiance (μW/cm^2^/s) and photon flux (μmol/m^2^/s photons), the latter was calculated with the Planck-Einstein relation (see S1 Table).

### Detection of transcript levels

Gene expression levels were determined by qPCR. To isolate RNA a metal bead (Peqlab Biotechnologie GmbH, Erlangen, Germany) and 350 μl RLT buffer (Qiagen N.V., Hilden, Germany) including 1% β-mercaptoethanol was added to the frozen larvae, which were homogenised for 3 min at 30Hz using a Qiagen tissue lyser followed by the Qiagen RNeasy mini protocol. 500 ng total RNA was used for each reaction to transcribe the RNA into cDNA with the QuantiTect reverse transcription kit (Qiagen). Intron spanning qPCR primers were designed with the universal probe library software from Roche, and qPCR reactions were performed using SYBR green PCR master mix from Applied Biosystems (Thermo Fisher Scientific Inc., Waltham, U.S.A.). *period1b* (*per1b*; NM212439, primer forward: 5’-gtcggatgatgacaaacagc-3’ and reverse: 5’-ccagagacacagacggacct-3’) and *clock1a* (*clk1a*; NM130957, primer forward: 5’-tcggaaactttaagtccctcaa-3’ and reverse: 5’-cactccctcaaagccgttt-3’) were used as a read-out for core clock gene oscillations, *period2* (*per2*; NM182857, primer forward: 5’-ccaacgtggacgaagatgta-3’ and reverse: 5’-gcagcaccttctggatgtct-3’) as a read out for a directy light regulated clock gene, and *thyrotroph embryonic factor α* (*tefα*; NM131400, primer forward: 5’-aaggcaataaatgaataatagtttgga-3’ and reverse: 5’- tcacctgcttctatcttgtctcc-3’) and *arylalkylamine N-acetyltransferase 2* (*aanat2*; NM131411, primer forward: 5’- ctggtggccttcatcattg-3’ and reverse: 5’- agagtccggcacgtgtgt-3’) to determine the effect on two genes that are under circadian clock control. The expression levels of the measured transcripts were normalized to *actin β* (*actb1*; NM131031, primer forward: 5’-tcactccccttgttcacaataa-3’ and reverse: 5’- ggcagcgatttcctcatc-3’), which does not show variation in diel expression levels [8]. To determine if the transcript levels between two conditions are significantly different the Student’s t-test [26] was applied.

## Results

### An instrument to control environmental conditions

#### General design

**Fig 1:**
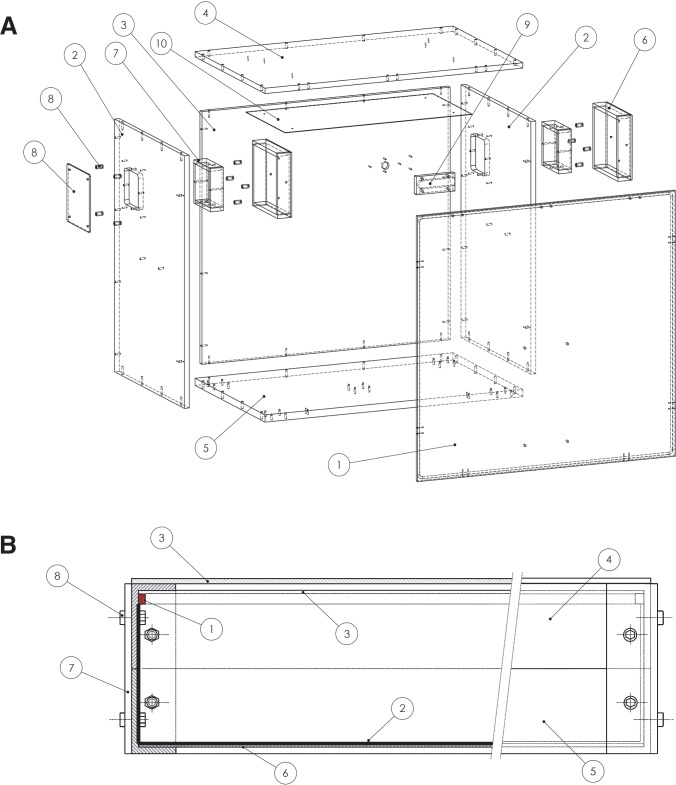
Construction plan for an instrument to control environmental conditions, and an infrared background lighting design to track small aquatic animals. (A) Technical drawing of the light-sealed temperature regulated instrument: 1) Front cover. 2) Side panels. 3) Rear panel. 4) Top panel. 5) Base. 6) Air vent cover. 7) Air vent flange. 8) Cap on air outlet. 9) Power supply cover. 10) Reflector plate (optional). For detailed technical drawings see S1 and S2 Figs. (B) Section through infrared light box (left) and outside (right), which is designed to emit a particularly even distribution of light. 1) IR light strip around the inside of the box. 2) White rigid projector screen on bottom and sides. 3) Two layers of opaque plexiglass separated by 3 mm. 4) Top aluminium frame. 5) Bottom aluminium frame. 6) Base. 7) Aluminium profiles on corners that connect the top and bottom frames. 8) Screws with nuts.

In order to keep environmental conditions constant, with the exception of the one under study, we designed an experimental setup that consists of a light-sealed ventilated chamber (Fig 1A and S1 Fig) containing a light source connected to a timer and a temperature controlled water tank. The instrument is constructed from matte black PVC to prevent reflection and damage from the exposure to humidity. The instrument consists of two side panels, a top and a base, one front cover and one rear panel (Fig 1A and S1 Fig). For the base a thick plate is used to support the weight of the equipment. The parts are assembled with hexagonal button flange head screws, and the edges are sealed with black silicone on the inside to prevent light from entering. The front cover is attached to the top panel with hinges so that the door can be folded up and does not obstruct the user while at work. A single cover is optimal for a simple and efficient light tight seal. The seal consists of a notch in the rim around the inside of the cover (S1 Fig) in which rubber tubing (Körner GmbH) is inserted so that the tubing presses against the rim around the chamber when the cover is closed. Two clips (Hettich GmbH) are attached to each side panel and to the base to secure the cover and apply pressure on the seal when closed. The cable inlet in the rear panel is shielded by a slotted cover on the inside to prevent light from entering (S1 Fig), and a cable junction box is fixed to the outside. An interchangeable light source is attached to the inside of the top panel and connected to an outside timer for light regime control. A 50 l rectangular white (for light reflection) plastic container is used as a temperature regulated tank (Linpac Stucki GmbH, Bad Salzufen, Germany). A Thermomix (B. Braun Biotech International GmbH, Melsungen, Germany) or Lauda Alpha (Lauda-Brinkmann LP, Delran, U.S.A.) immersion thermostat is attached to the tank (S1 Fig), as these thermostats are capable of maintaining a constant water temperature within the ± 0.1°C range. The Lauda thermostat can also be equipped with a dedicated cooling unit to cool or generate temperature cycles. Since the display of the thermostat emits light the top part of the thermostat should be covered with blackout material (e.g. aluminium foil). Ventilation is essential to keep the temperature in the instrument constant and to avoid condensation. For ventilation the instrument is equipped with a fan behind the air inlet in one side panel and an air outlet in the opposite side panel. Importantly, to create an air flow through the closed instrument while preventing the light from entering, the air in and outlets are covered with a maze construction (Fig 1A and S1 Fig). To further block light from entering the instrument a fan with scoops (AC centrifugal compact fan, ebm-papst RG 90-18/56) instead of blades is installed behind the air inlet. Since the air outlet does not contain a fan, an extra cover to prevent light from entering is placed on the outside (Fig 1A and S1 Fig). Wheels and handles are attached to the instrument to facilitate movement. The sample holder is designed to keep the samples submerged in water, and consists of a cover connected to a base, its cover is made of transparent plexiglas and has holes for optimal light transmission, and the base has feet to keep the samples off the ground so that water can flow under the samples.

#### Light sources

**Fig 2:**
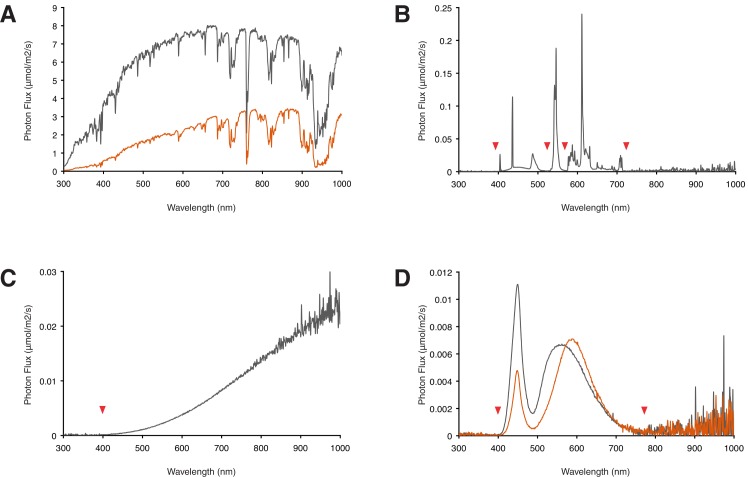
Spectra of sunlight and artificial light sources. For light dependent processes the photon flux has a higher relevance than the irradiance since photoreceptors are activated by photons, thus we compared spectra plotted in μmol/m^2^/s photons versus the wavelength. (A) Spectrum of sunlight on a cloudless summer day in Vienna at 12:00 hours in grey (22437 μmol/m^2^/s photons) and at 19:00 hours during sunset in orange (1268 μmol/m^2^/s photons). (B) Spectrum of a Philips compact fluorescent lamp (14W, 5.1 μmol/m^2^/s photons). Red arrowheads indicate gaps in the spectrum. (C) Spectrum of an Osram incandescent lamp (15W, 5.8 μmol/m^2^/s photons). (D) Spectra of a Winger white daylight LED (1W, 6500K, 1.4 μmol/m^2^/s photons) in grey, and warm white LED (1W, 3200K, 1.1 μmol/m^2^/s photons) in orange. The main difference between a daylight and a warm white LED is the light intensity around 450 nm. Note the similarity of these light sources to respectively daylight and twilight in A. All measurements except for sunlight were performed at a distance of 60 cm from the light source, which corresponds to the distance between light source and sample in our experimental instrument.

The light source represents a key element of the instrument. The importance of carefully considering its spectrum and intensity is exemplified by the fact that different qualities of light have been demonstrated to differentially impact on zebrafish growth and development [27]. There are two principles for choosing a light source: Either light covering a broad range of the spectrum, i.e. “white light”, is used, or the light source is wavelength-specific (e.g. to study a specific photoreceptor). Which “white light” should be chosen? From ecological and evolutionary considerations a light source that generates a broad light spectrum resembling that of sunlight (Fig 2A) would be a logical choice. However, it is complicated to define natural light as the intensity and spectrum of sunlight below the earth’s atmosphere vary enormously, and depend on the latitude, the season, the weather, and on the the time of the day (Fig 2A). Furthermore, the water depth and turbidity will selectively filter wavelengths [28]. Nevertheless, ideally an artificial light source should have a continuous spectrum so that photoreceptors from a range of absorption optimums will all receive the optimal wavelengths of light. To choose the appropriate light source for the study of light dependent processes, we compared the photon flux (μmol/m^2^/s) of the spectra instead of the irradiance (μW/cm^2^/s) as photoreceptors are activated by photons. The compact fluorescent lamp (CFL) shows large differences in light intensity over the visible spectrum (compare Fig 2B with 2A) and gaps are present. An incandescent (Tungsten) lamp emits light over the whole visible spectrum with a higher intensity in the red part of the spectrum than the blue (Fig 2C), its light distribution thus resembling that of twilight. The emission of white light emitting diodes (LED) covers the visible spectrum and shows only gaps in the UV and IR (Fig 2D), thus this continuous spectrum is suitable for a wide range of studies. Importantly, a standard white light LED has a colour temperature (6500K) similar to that of daylight while a warm white LED mimics twilight (compare Fig 2D with Fig 2A). The high clarity and relatively low water depth used in the setup prevents significant alterations in the intensities of the emitted wavelengths.

**Fig 3:**
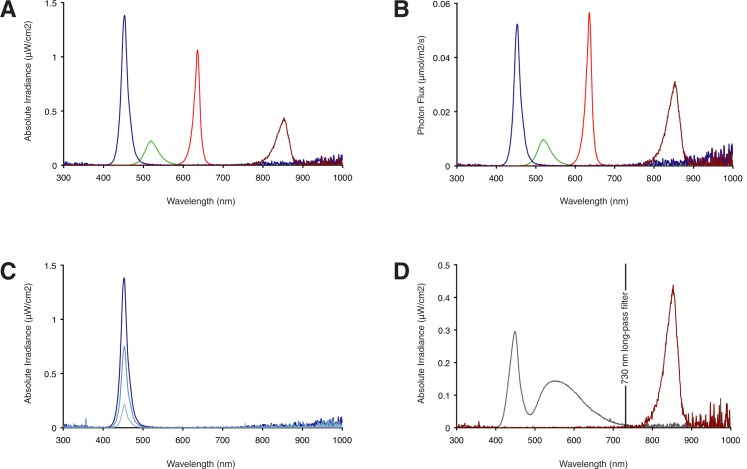
Light sources with narrow bandwidth spectra. For comparison all spectra were measured from LEDs (Winger Electronics GmbH) of the same manufacturer near their maximum power (1W, 340 mA) and at a distance of 60 cm in air. (A) Spectrum of a blue LED (39 μW/cm^2^,1.7 μmol/m^2^/s photons), green LED (11 μW/cm^2^, 0.5 μmol/m^2^/s photons), red LED (23 μW/cm^2^, 1.2 μmol/m^2^/s photons), and IR LED (20 μW/cm^2^, 1.4 μmol/m^2^/s photons). (B) The same spectra as in A, with instead of absolute irradiance the photon flux plotted on the y-axis. Note when comparing two LEDs that emit at different wavelengths but produce similar absolute irradiance (compare blue with IR), that the photon flux of the photons with a larger wavelength will be higher. (C) Reducing the intensity narrows the bandwidth of the spectrum. Compare blue LED driven at a current of 340 mA (39 μW/cm^2^, 1.69 μmol/m^2^/s photons) in dark blue with the same LED at 170 mA (21 μW/cm^2^,0.94 μmol/m^2^/s photons) and at 50 mA (6 μW/cm^2^, 0.29 μmol/m^2^/s photons) in light blue. This chart shows that the light intensity affects the bandwidth of the spectrum disproportionally. Also note the near linear correlation between the current and the light intensity. (D) A long-pass filter (Lee filter #87) is inserted between the camera and the lens to block visible light below 730 nm. Note that the spectra of the IR LED (red) and the white LED (grey) are on opposite sides of the filter. As the white LED does not emit IR, a LD cycle will not produce intensity fluctuations in the recording and thereby affect the tracking.

Which considerations should be made when choosing light sources with a specific narrow spectrum? With many Opsins present in fish and other aquatic organisms, redundancy may be an obstacle when studying the function of a particular photoreceptor or group of photoreceptors. An approach to circumvent this problem is to use a light source that emits in a narrow band of the spectrum where the photoreceptor under study has its absorption optimum, thereby avoiding activation of photoreceptors that exhibit an absorption optimum far outside that range. Furthermore, to narrow down which wavelengths and/ or photoreceptors play a role in a particular process one can use different light sources that emit in distinct parts of the spectrum [27]. For instance, blue light is sufficient for entraining the zebrafish circadian clock [14, 15]. LED technology has the advantage that a light source can be chosen which emits a spectrum with narrow bandwidth fitting the process under study (Fig 3A). Note that the green LED in Fig 3A produces a lower light intensity than the blue and red LEDs due to a lower efficiency in converting electrical power into light. When we compare the photon flux with the energy of different spectra from LEDs (Fig 3A and 3B), such as the blue and IR LEDs, we observe that although the IR LED produces only half the absolute irradiance (19.76 μW/cm^2^) of the blue LED (39.09 μW/cm^2^) it emits nearly as many photons (respectively 1.39 μmol/m^2^/s and 1.69 μmol/m^2^/s, Fig 3B). Thus the higher number of photons from an IR light source could potentially compensate for the inefficiency of activating a photoreceptor with an absorption that only partially overlaps with the IR spectrum. Importantly, the width at the base of the curve of the blue, green, red and infrared LEDs (Fig 3A) shows that the emitted light covers a considerable part of the spectrum. Note that although the light intensity has a near linear correlation with the current, the bandwidth of the experimental spectrum is affected disproportionally (Fig 3C).

LEDs have the advantage that a light source, which emits a specific spectrum, can be selected and the light intensity can be regulated. To install LEDs (Winger Electronics GmbH, Dessau-Roßlau, Germany) mount them on cooling blocks (37 mm x 33 mm x 10 mm, Winger Electronics GmbH) using heat conducting metal glue (Arctic Alumina, Winger Electronics GmbH) to facilitate the release of heat. For attachment a 6 mm hole is drilled and tapered into the middle of the cooling block, a 6M screw (length 2 cm) with a countersunk head and a flat top is glued in the hole, on top of which the LED is glued. Nylon washers and a low-profile T-nut (Thorlabs Inc. Newton, U.S.A.) are placed on the screw, thereafter the LED on the cooling block is attached to a 25 mm (length 37 cm) optical construction rail (Thorlabs Inc.). We wired three or four, 1W or 3W, LEDs in series (depending on the photon flux of the LEDs) and four of these sets in parallel. Since the current stays the same through any component in a series circuit and voltage stays the same over all components in a parallel circuit, the number of these sets depends on the power of the LEDs and the maximal output of the power supply. The light intensity should be set by driving the LEDs with the current (Fig 3C), as a small change in voltage produces a large change in current and thus in the light intensity. The optical rails with LEDs are attached to the ceiling of the instrument with screw and nut, and can be easily exchanged by sliding them out.

#### Recording with IR light and tracking

The instrument can be further adapted for behavioural studies. For analysis of activity levels an industrial camera (acA2040-90um, Basler AG, Ahrensburg, Germany) is installed and visible light is blocked by inserting a 730 nm long-pass filter (LeeFilter, Infrared polyester #87) (Fig 3D). A lens with the optimal focal length (http://www.baslerweb.com/en/support/tools/lens-selector) is attached to the camera (Kowa, 25mm or 16mm F1.4). The lens should be compatible with the pixel size of the camera and should not block IR light (SWIR type).

An even distribution of background light is crucial for high quality tracking. To achieve this we designed a box (Fig 1B, S2 Fig) in which IR light is reflected by a projector screen on the bottom and the out going indirect light is scattered by opaque plexiglas on the top. The IR light box consists of eight unequal L-shaped aluminium profiles (3.0 cm x 5.0 cm). Each profile is cut on both short sides in a 45° angle (35.8 cm x 35.8 cm), and four profiles are glued (Arctic Alumina) together to form a square frame. The two frames are placed on top of each other so that the longest sides of the L stand on each other and the shortest form the top and bottom. Four equal L-shaped (3.0 cm x 3.0 cm) aluminium profiles of 10.0 cm length are secured on the outside corners with 6M hexagonal flange head screws (10 mm length) and nuts to connect the frames and provide support. A rigid ProWhite projector screen (Carl’s Place, Milton, U.S.A.) is glued to a plexiglas base, which is fixed to the bottom frame with double sided construction tape. Two opaque plexiglas plates, OM200 SC and 7D007 RP (Evonik Industries AG, Essen, Germany) are glued to respectively the outside and inside of the top frame. A strip of IR LEDs (840 nm Solarox, Winger Electronics GmbH) is glued in the corner along the sides of the top frame. This design releases the heat from the IR LEDs to the sides instead of below the animals, which is an advantage. A hole is drilled on one corner for the outlet of electric cables. For locomotor experiments each animal is placed in a well of a temperature controlled tracking plate (S2 Fig). This is a 32 cm (width) x 32 cm (length) x 2 cm (height) transparent sanded acrylic plate with an array of 5 by 5 concave wells (diameter: 34 mm and depth: 17 mm) milled in the top, and a 3 mm grove milled 1.5 cm from the rim in the bottom. A silicone seal is placed in the grove. The plate with wells is placed on top of a plate from the same size and material that has the center part, except for the 25 mm wide rim, milled out (depth: 17 mm) and that has two in- and outlet holes on opposite sides for the flow of temperature controlled water under the tracking plate. The plates are connected with 4M screws. The IR light box is placed under the tracking plate to avoid shadows from the fish and reflections from the water.

The open source frame grabber software VirtualDub (http://sourceforge.net/projects/virtualdub/) can be used for recording as it is compatible with Pylon (the interface software provided with the camera), and frames can be saved in MPEG or AVI format. The movies can be tracked using free tracking software (www.idtracker.es/) [29] or the commercially available tracking software EthoVision from Noldus Information Technology B.V. (Wageningen, The Netherlands) or software from loopbio (Kritzendorf, Austria).

### Testing infrared light effects on the zebrafish circadian clock

In order to demonstrate the method and to illustrate the features of the instrument while providing an important control for reliable experimental conditions, we have followed our experimental procedure to test the impact of IR light on the circadian clock of the zebrafish.

**Fig 4:**
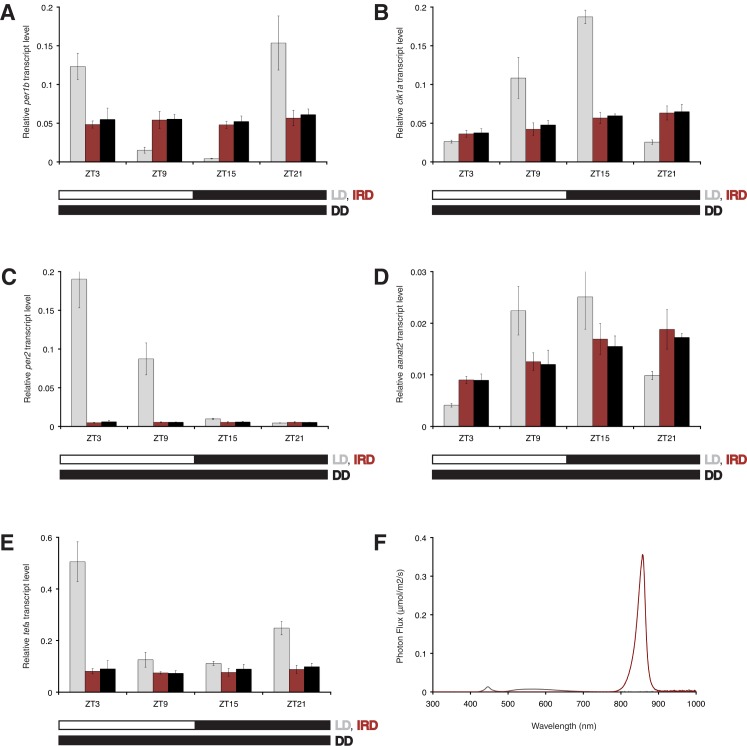
IR light does not entrain the zebrafish circadian clock. The transcript levels of three core circadian clock genes and two clock output genes in larvae exposed to six 12–12 hour white light-dark or IR light-dark cycles and larvae raised in constant darkness, all at 28.0 ± 0.2°C, were used as readout. (A) *per1b* (circadian clock negative feedback loop) transcript levels are compared between larvae kept in constant darkness (DD, black bars) and under an IR light-dark (IRD, red bars) and white light-dark regime (LD, grey bars). The white-black bar below the chart indicates the light-dark interval and the black bar indicates constant darkness. (B) *clk1a* (circadian clock positive feedback loop), (C) *per2* (circadian clock directly light regulated), (D) *aanat2* (circadian clock output) and (E) *tefα* (circadian clock output) transcript levels under the same conditions as in A. None of the analyzed genes exhibit significant differences in transcript level between the IRD and DD conditions for each ZT (p>0.05, two-sample equal unpaired Student’s t-test). Error bars: standard deviations. (F) Intensities of IR light (154 μW/cm^2^, 10.96 μmol/m^2^/s photons) in red and white daylight (33 μW/cm^2^, 1.57 μmol/m^2^/s photons) in grey to which the larvae were exposed in this experiment.

Behaviour is often used as a readout in neuroscience, including studies of photoreceptors and chronobiology. For behavioural recordings IR light (Fig 3F) is commonly used as it allows activity to be documented in the absence of visible light. While most vertebrate photoreceptors contain the light absorbing chromophore retinal, many fish, amphibians and reptiles use 3,4-didehydroretinal, which allows them to detect longer wavelengths further into the red. In addition, visible red light has been demonstrated to induce clock gene expression in zebrafish [15]. Thus IR light might potentially act as a zeitgeber. We assessed whether IR light can set the circadian clock, and thus significantly impact on behaviour, metabolism and physiology. Wildtype zebrafish larvae were exposed for 6 days to a 12–12 hour infrared light-dark (IRD) regime and compared with larvae kept in constant darkness (DD) and larvae exposed to a 12–12 hour white light-dark (LD) regime, all maintained at a constant temperature of 28.0 ± 0.2°C. Samples were taken at four time points separated by 6 hour intervals. We analysed the expression levels of five genes: the core circadian clock genes *per1b* and *clk1a*, part of the negative and positive transcription-translation feedback loop respectively, the directly light regulated circadian clock gene *per2*, which had been shown to exhibit rhythmic transcript levels under a red LD cycle [15], and the genes *tefα* and *aanat2*, which are under circadian clock control [30]. The transcript levels of *per1b* under IRD conditions do not show a significant difference with the levels under DD (ZT3; p = 0.53, ZT9; p = 0.83, ZT15; p = 0.43, ZT21; p = 0.54, equal unpaired Student’s t-test), which are both non-rhythmic intermediate levels when compared to the peak and trough levels of robust gene expression cycling under a LD regime (Fig 4A). Also the transcript levels of *clk1a* do not show a significant difference between IRD and DD conditions (Fig 4B) at each ZT (ZT3; p = 0.78, ZT9; p = 0.39, ZT15; p = 0.52, ZT21; p = 0.83, equal unpaired Student’s t-test). Consistently, also *aanat2* (ZT3; p = 0.90, ZT9; p = 0.75, ZT15; p = 0.57, ZT21; p = 0.60, equal unpaired Student’s t-test) and *tefα* (ZT3; p = 0.74, ZT9; p = 0.80, ZT15; p = 0.39, ZT21; p = 0.43, equal unpaired Student’s t-test) do not show a significant difference in transcript levels at each ZT between the IRD and DD conditions (Fig 4D and 4E). As reported previously [12, 31] we observe a non-rhythmic increase in transcript levels during development for *clk1a* and *aanat2* (ZT3 vs ZT21 equal unpaired Student's t-test *clk1a* IRD: p = 0.00763, *clk1a* DD: p = 0.00979, *aanat2* IRD: p = 0.00548, *aanat2* DD: p = 0.00140). In contrast to the LD regime overall levels of *per2* are low under IRD and DD conditions with no significant difference in transcript levels between these conditions (ZT3; p = 0.27, ZT9; p = 0.82, ZT15; p = 0.57, ZT21; p = 0.93, equal unpaired Student’s t-test), thus this directly light controlled gene is not induced by IR light (Fig 4C).

The transcript levels of *per1b*, *clk1a*, *per2*, *tefα and aanat2* under an IRD regime are in the same range as the levels observed in DD, which implies that the zebrafish circadian clock is not set by 840 nm IR light. We interpret the arrhythmicity under DD and IRD to reflect the presence of multiple circadian oscillators that are out of phase and are not set by light [12, 13, 32–34]. Importantly, these data also demonstrate that the instrument presented here, when operated under constant darkness and constant temperature, eliminates all stimuli that set the circadian clock.

## Discussion

With the instrument presented here one can test the effect of specific light and temperature parameters on biological processes by chosing wavelength, light intensity and temperature on a range of aquatic organisms. A temperature controlled environment is a crucial feature of any setup when studying the effect of light on aquatic ectotherms, which are easily affected by environmental temperature fluctuations [23] as they lack homeostatic body temperature control and also lose body heat much more rapidly than terrestrial animals due to the high heat capacity of water [35]. It is therefore not surprising that daily temperature fluctuations of ± 1.0°C have been demonstrated to entrain the zebrafish circadian clock [22]. In additon to preventing temperature fluctuations our design also eliminates light contamination. This can be tested by developing a film which has been inside the closed setup while the light inside the setup was off and outside the setup was switched on. We found that not all commercially available setups are light-tight. This is problematic since even short exposure to low intensity visible light can re-set the circadian clock [13]. As IR light is used in many studies, it is crucial to determine if it affects the circadian clock. A previous study showed that optokinetic motor response, which fully depends on vision, is not affected by IR [20]. However so far the effect of IR light on non-visual processes has not been assessed, even though red light has been shown to entrain the zebrafish circadian clock [15], and the tail of the IR LED spectrum reaches below the 800 nm (Fig 3A and 3B). We addressed this issue by exposing zebrafish larvae to an IRD cycle instead of constant IR light and by using high intensity IR light thereby increasing the IR under 800 nm (Fig 4F). We here show that IR light does not induce a rhythm in the transcript levels of the core circadian clock genes *per1b*, *clk1a*, *per2* and the clock output genes *aanat2* and *tefα* (Fig 4A–4E). We conclude that 840 nm IR light sources can be used to study the zebrafish circadian clock.

Since food intake also acts as a zeitgeber [36, 37], this environmental cue can interfere with other zeitgebers and should be controlled to minimize its impact when studying light-dependent processes. We discuss some of its known effects here in order to manipulate the impact of this zeitgeber. Zebrafish larvae do not require food until the sixth day post fertilisation when kept at 28°C. Obviously, this advantage is partially lost when studying later stages, and in this case the strength of the zeitgeber depends on the biological process under study and the timing of food administration. For instance in Medaka, feeding entrains a circadian rhythm of agonistic behaviour, which continues through a fasting period of three days. However, the daily rhythms of mating stay fixed to the light-dark cycle and are not phase-shifted by the feeding time [38]. The differential effects of these zeitgebers indicate that these behaviours are linked to different parts of a multi-oscillator system that is loosely connected or may not be connected at all [39]. Thus food as a zeitgeber may set only a subset of clocks in specific organs and tissues. Although it has been demonstrated that the timing of food availability entrains the circadian clock [40], a separate food entrainable oscillator could exist [41]. The differential response to random feeding and feed-fast cycles demonstrates that the strength of this zeitgeber depends on its timing. In gold fish, the feeding associated cortisol rhythms are entrained to the light-dark cycle when the animals are held on a random feeding regime [42]. However, this rhythm becomes independent of the light-dark cycle and entrained by food when the animals are fed a single meal at a fixed time of the day [43]. Thus, when the effect of food as a zeitgeber is not desired, it is an option to restrict research to (zebrafish) larval stages. Alternatively, random feeding and/or feeding on alternate days will reduce its effect.

Here we have presented an optimized instrument and protocol for photo- and chronobiology research, and demonstrated its application for aquatic organisms. Together with the knowledge how to manipulate the relative strength of non-photic zeitgebers we have introduced an affordable multi-purpose tool for reliable molecular and behavioural data acquisition.

## Supporting information

S1 Fig Drawings of instrument for photobiology. (PDF)

S2 Fig Drawings of infrared light box and tracking plate. (PDF)

S1 Table Tables of spectra. (XLS)
