# Supplementary material for: Instrument design and protocol for the study of light controlled processes in aquatic organisms, and its application to examine the effect of infrared light on zebrafish
Source: PLoS One. 2017 Feb 17;12(2):e0172038. doi: 10.1371/journal.pone.0172038 (PMC5315407; doi:10.1371/journal.pone.0172038)
Supplement: S1 Fig — (PDF) [file pone.0172038.s001.pdf]

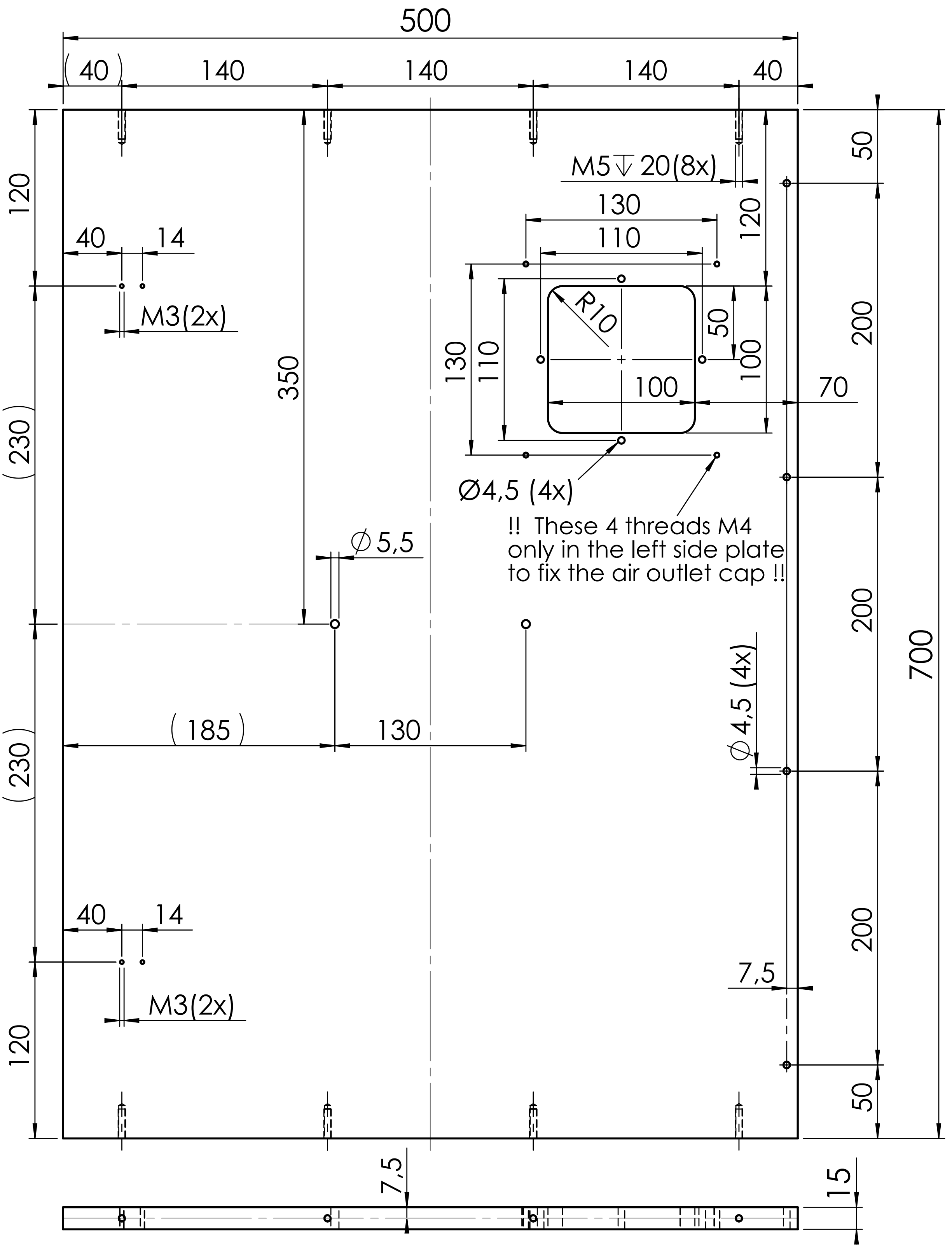

Toleranz: DIN 7168-m	Oberfläche:	Gratfrei ohne scharfe Kanten<br>Menge: 2	EMBL Heidelberg<br>European Molecular Biology Laboratory	
	Name	Datum	Zeichen	
gez.:		2014		
gepr.:				Werkstoff: PVC schwarz
freigeg.				Gewicht:

Projekt:	Instrument for photo- & chronobiology	Konto:
Entwerfer:	Marcus P.S. Dekens	
Zeichnung:	Side panel	A4
Maßstab 1:10		Blatt 1 von 1

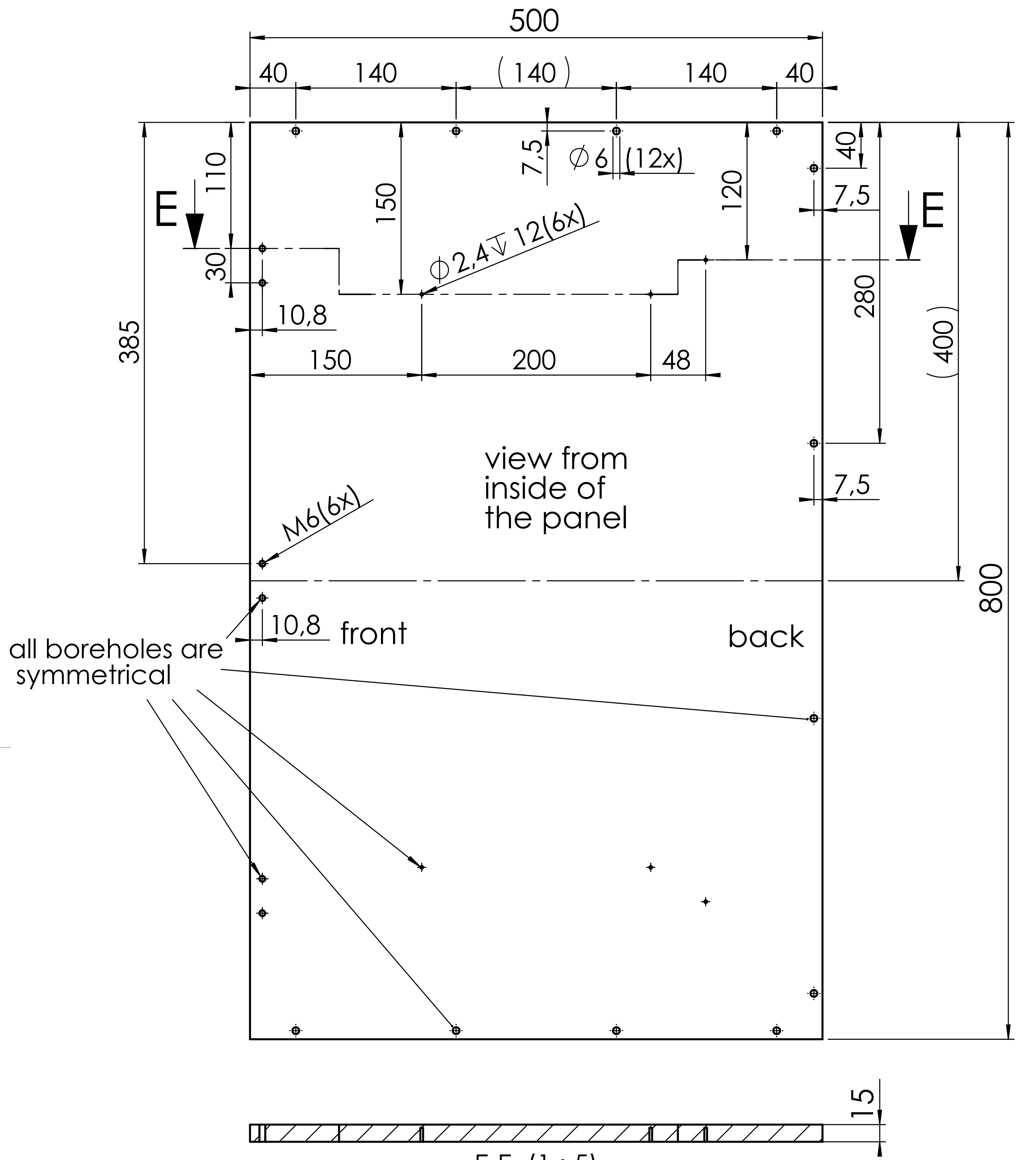

E-E (1 : 5)

Toleranz: DIN 7168-m	Oberfläche:	Gratfrei ohne scharfe Kanten<br>Menge: 1	EMBL Heidelberg<br>European Molecular Biology Laboratory

	Name	Datum	Zeichen			
gez.:		2014			Projekt: Instrument for photo- & chronobiology	Konto:
gepr.:				Werkstoff: PVC schwarz	Entwerfer: Marcus P.S. Dekens	
freigeg.					Zeichnung: Top panel	A4
				Gewicht:	Maßstab 1:10	Blatt 1 von 1

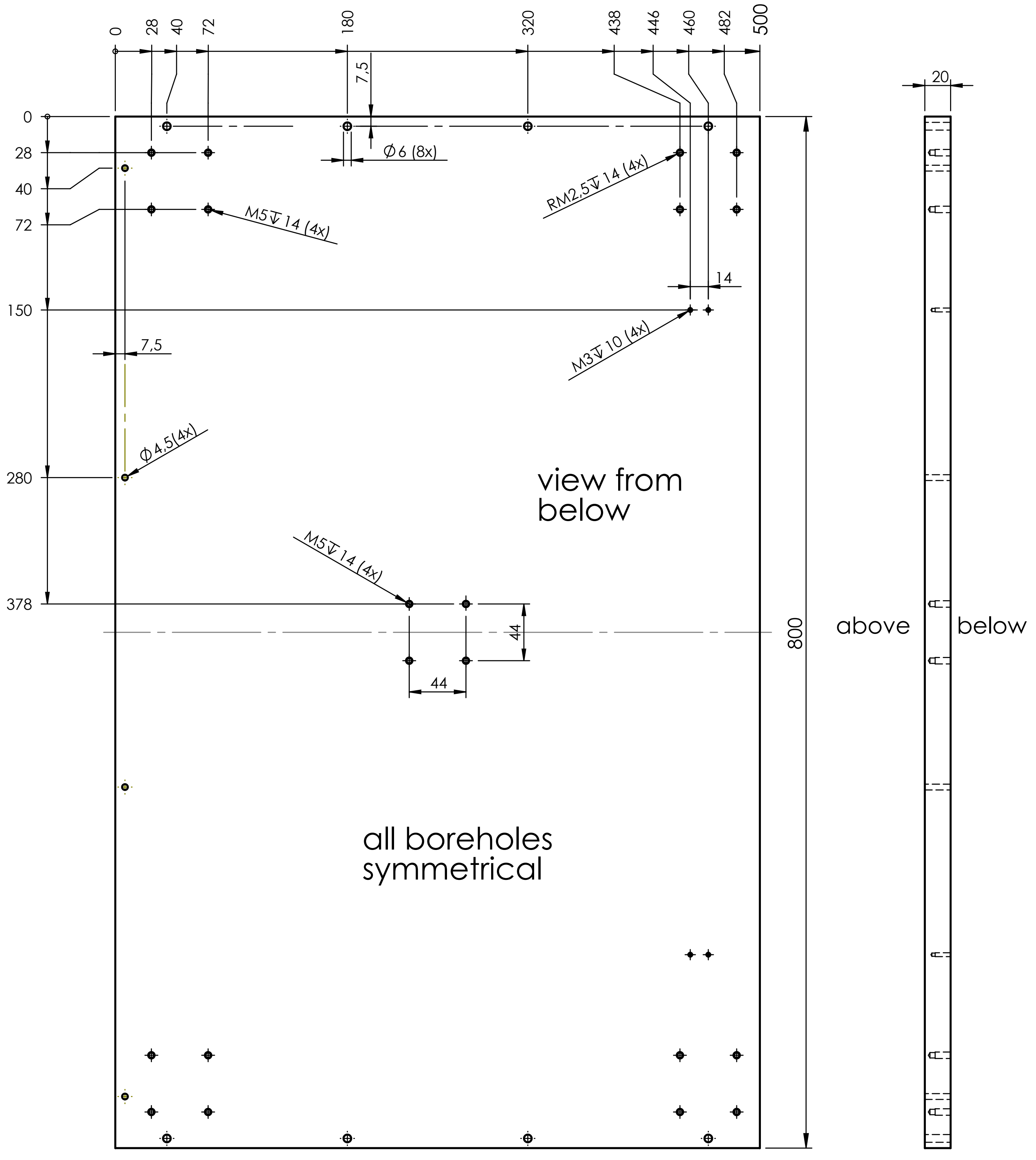

Toleranz: DIN 7168-m

Oberfläche:

Gratfrei ohne scharfe Kanten

Menge: 1

EMBL Heidelberg
European Molecular Biology Laboratory

	Name	Datum	Zeichen
gez.:		2014	
gepr.:			
freigeg.			

Werkstoff: PVC black

Gewicht:

Projekt: Instrument for photo- & chronobiology

Konto:

Entwerfer: Marcus P.S. Dekens

Zeichnung: Base

A4

Maßstab 1:10

Blatt 1 von 1

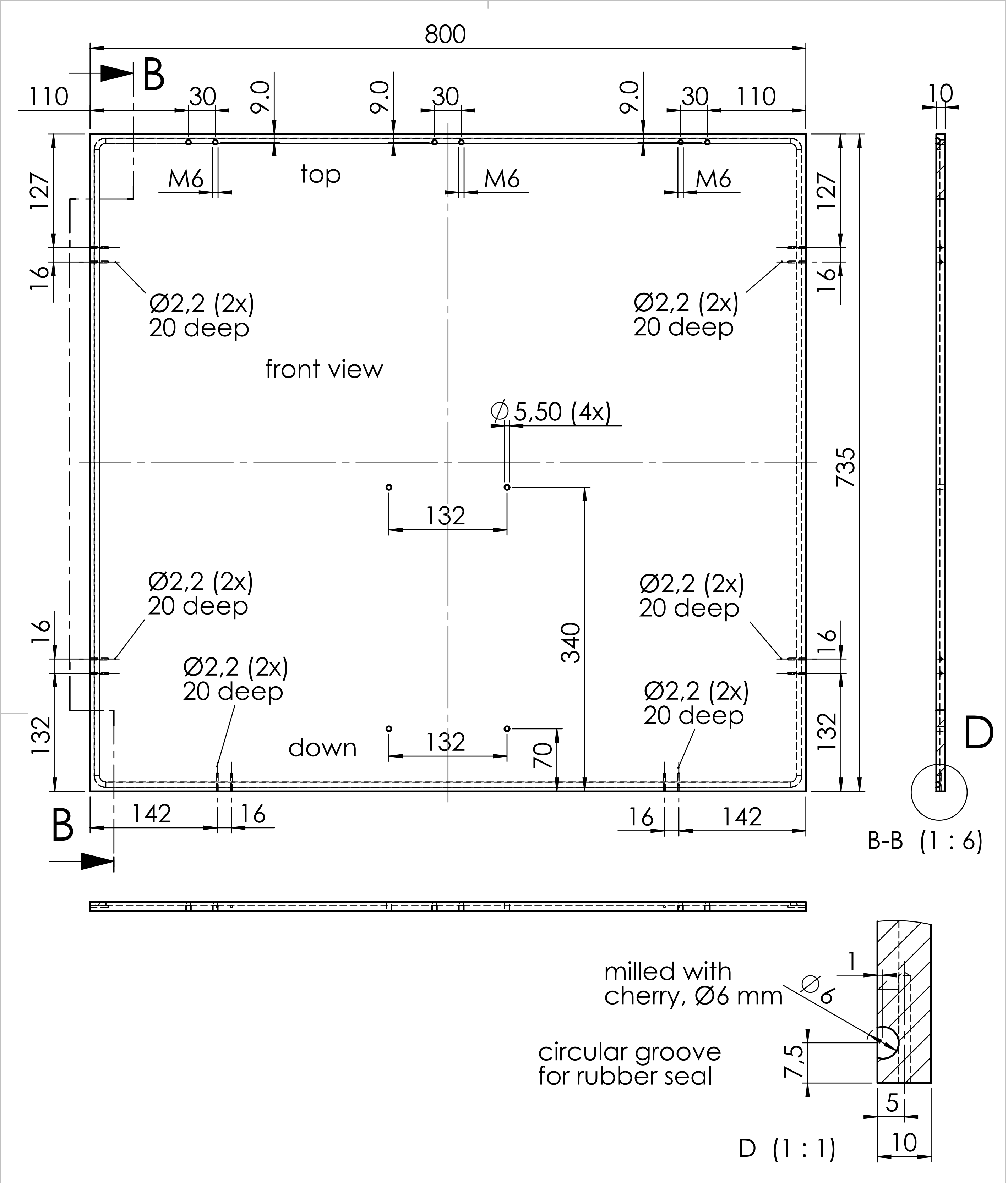

Toleranz: DIN 7168-m	Oberfläche:	Gratfrei ohne scharfe Kanten<br>Menge: 1	EMBL Heidelberg<br>European Molecular Biology Laboratory

	Name	Datum	Zeichen		
gez.:		2014		Projekt:	Instrument for photo- & chronobiology (Konto:)
gepr.:				Werkstoff: PVC black	Entwerfer: Marcus P.S. Dekens
freigeg.					Zeichnung: Front cover — A4
				Gewicht:	Maßstab 1:10 — Blatt 1 von 1

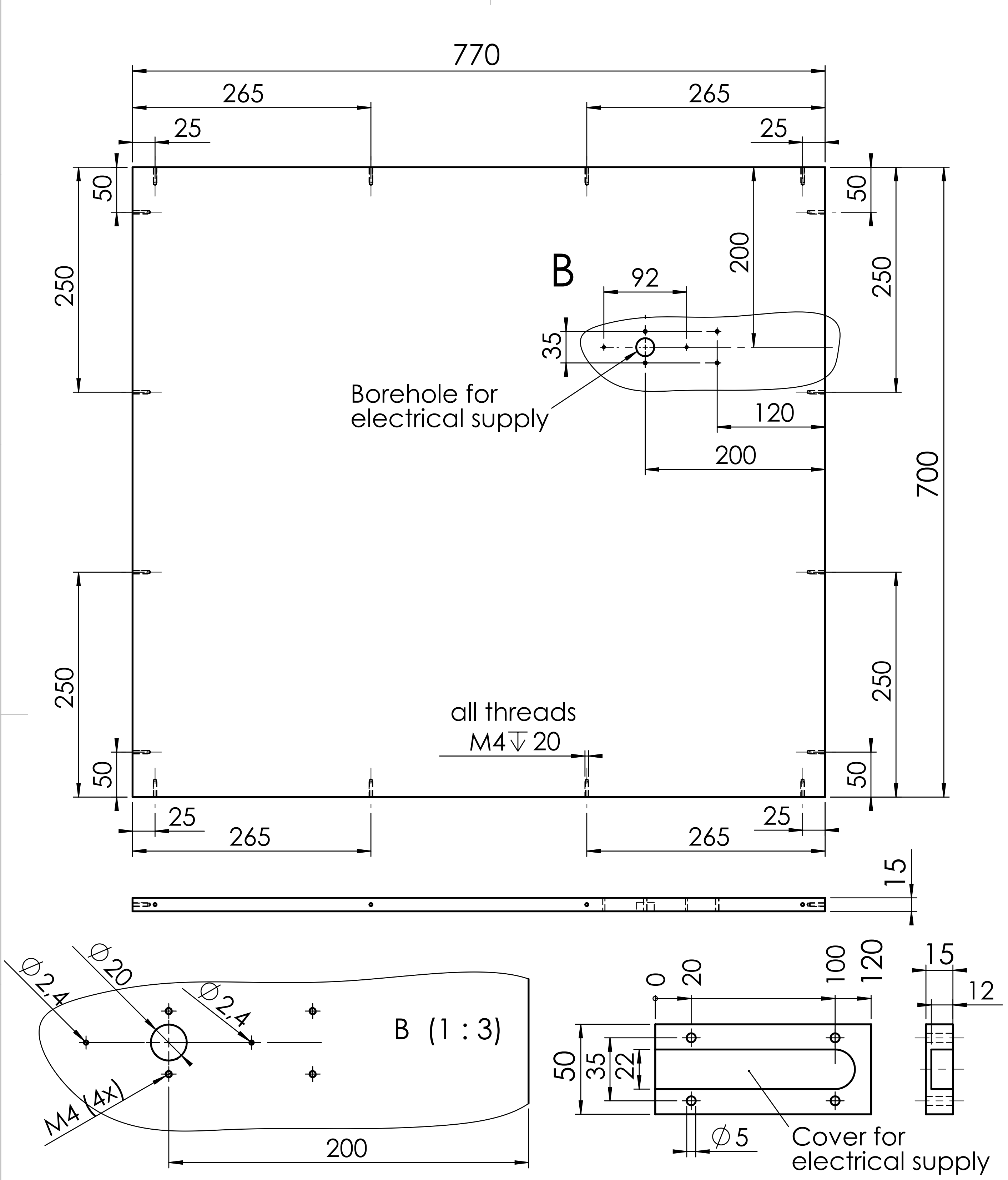

Toleranz: DIN 7168-m	Oberfläche:	Gratfrei ohne scharfe Kanten	EMBL Heidelberg European Molecular Biology Laboratory
Stand:		Menge: 1	

	Name	Datum	Zeichen		
gez.:		2014			Projekt: Instrument for photo- & chronobiology
gepr.:				Werkstoff: PVC black	Entwerfer: Marcus P.S. Dekens
freigeg.					Zeichnung: Rear Panel
				Gewicht:	Maßstab 1:10

Konto:

A4

Blatt 1 von 1

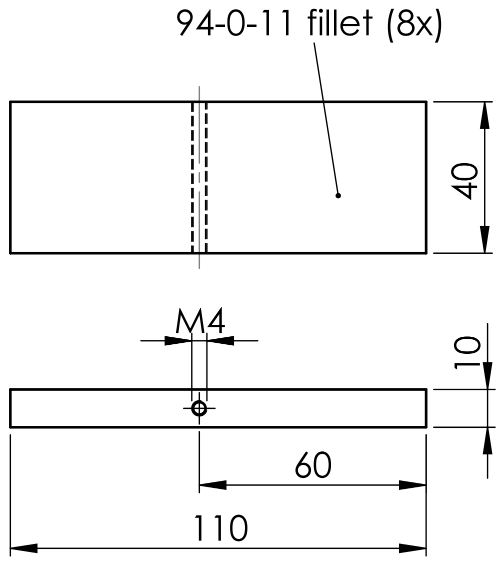

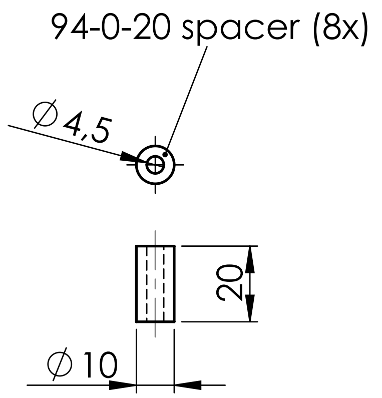

for 1 setup 2 complete flanges are required.

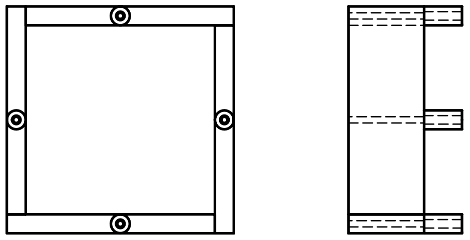

fillets glued with "Tangit PVC-glue", pretreatement with "Tangit PVC-cleaner". (Tangit products made by HENKEL, Germany)

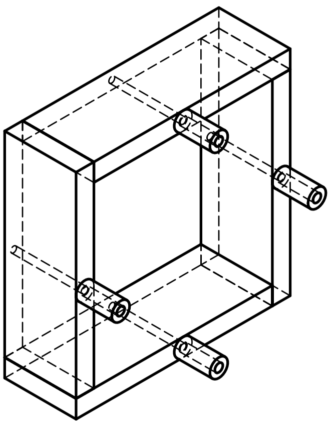

Toleranz:	DIN 7168-m
Oberfläche:	
Gratfrei ohne scharfe Kanten	
Menge:	2 units
gez.: Datum	2014
gepr.:	
freigeg.	
Werkstoff:	PVC black
Gewicht:	
Projekt:	Instrument for photo- & chronobiology
Entwerfer:	Marcus P.S. Dekens
Zeichnung:	Air vent flange
Konto:	
Format	A4
Maßstab	1:10
Blatt	1 von 1

EMBL Heidelberg
European Molecular Biology Laboratory

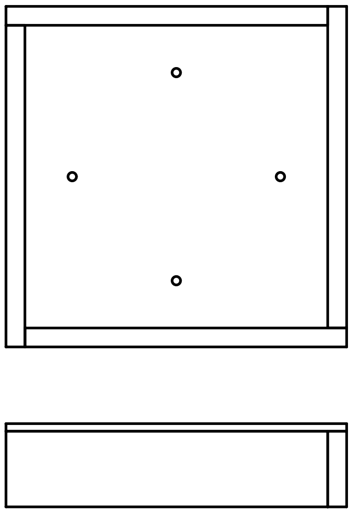

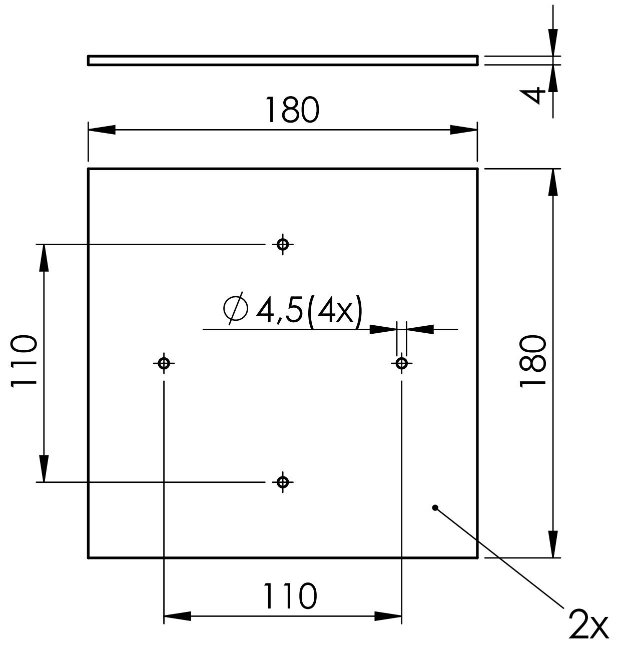

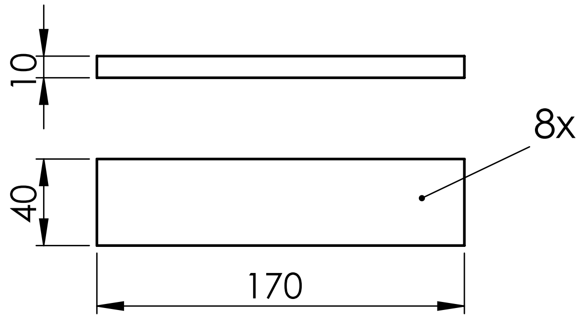

for 1 setup 2 complete
cover caps are required.

all parts glued with "Tangit PVC-glue",
pretreatement with "Tangit PVC-cleaner".
(Tangit products made by
HENKEL, Germany)

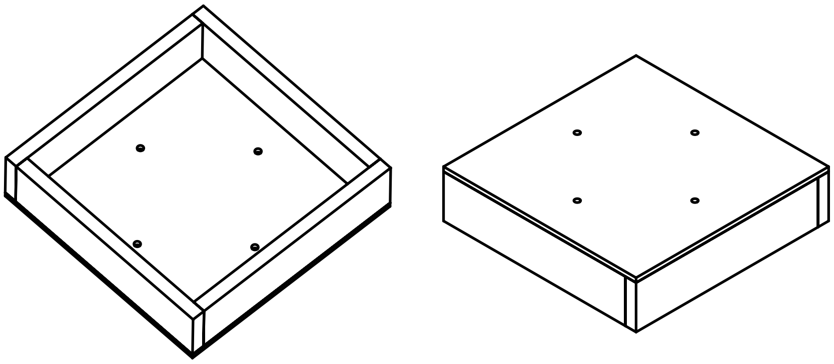

Toleranz:	DIN 7168-m
Oberfläche:	
Gratfrei ohne scharfe Kanten	
Menge:	2

EMBL Heidelberg
European Molecular Biology Laboratory

	Name	Datum	Zeichen
gez.:		2014	
gepr.:			
freigeg.			

Werkstoff:	PVC black
Gewicht:	
Projekt:	Instrument for photo- & chronobiology
Konto:	
Entwerfer:	Marcus P.S. Dekens
Zeichnung:	Air vent cover
	A4
Maßstab	1:10
Blatt 1 von 1	

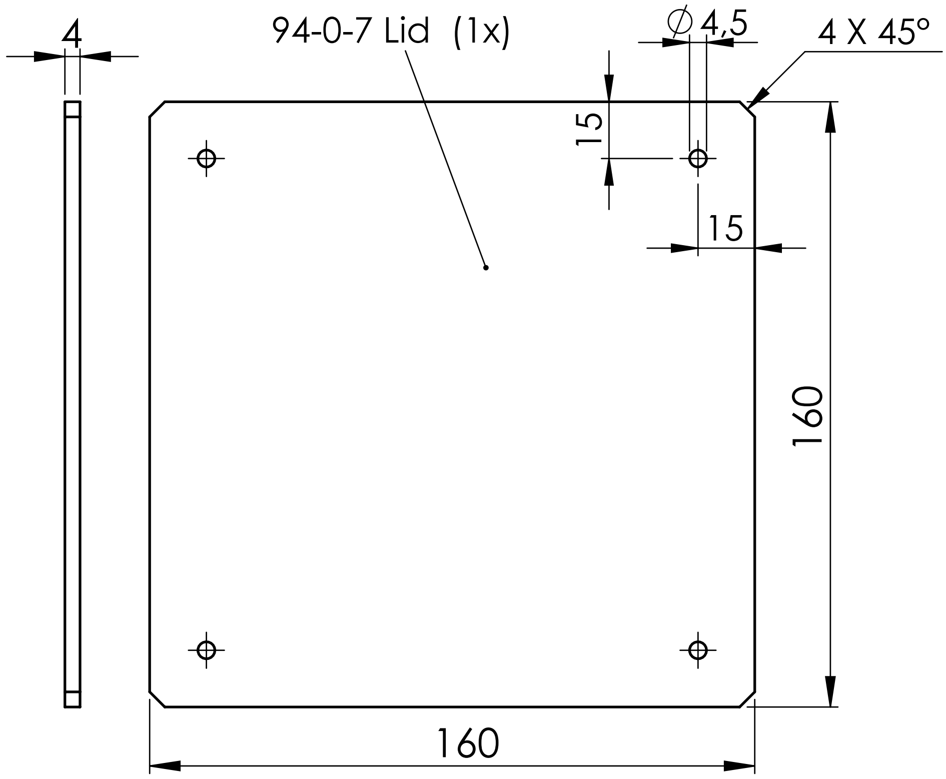

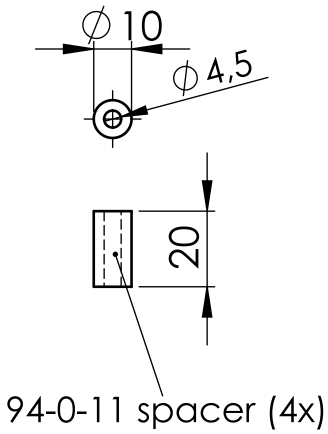

1 lid for air vent with
4 spacers fixed on the
left side plate.

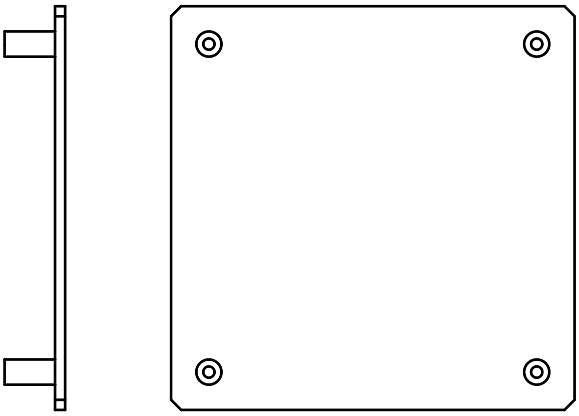

all parts glued with "Tangit PVC-glue",
pretreatement with "Tangit PVC-cleaner".
(Tangit products made by
HENKEL, Germany)

Toleranz: DIN 7168-m

Oberfläche:

Gratfrei ohne scharfe Kanten

Menge: 1 unit

EMBL Heidelberg
European Molecular Biology Laboratory

	Name	Datum	Zeichen
gez.:		2014	
gepr.:			
freigeg.			

Werkstoff: PVC black

Gewicht:

Projekt: Instrument for photo- & chronobiology

Konto:

Entwerfer: Marcus P.S. Dekens

Zeichnung: Air outlet cap

A4

Maßstab 1:10

Blatt 1 von 1

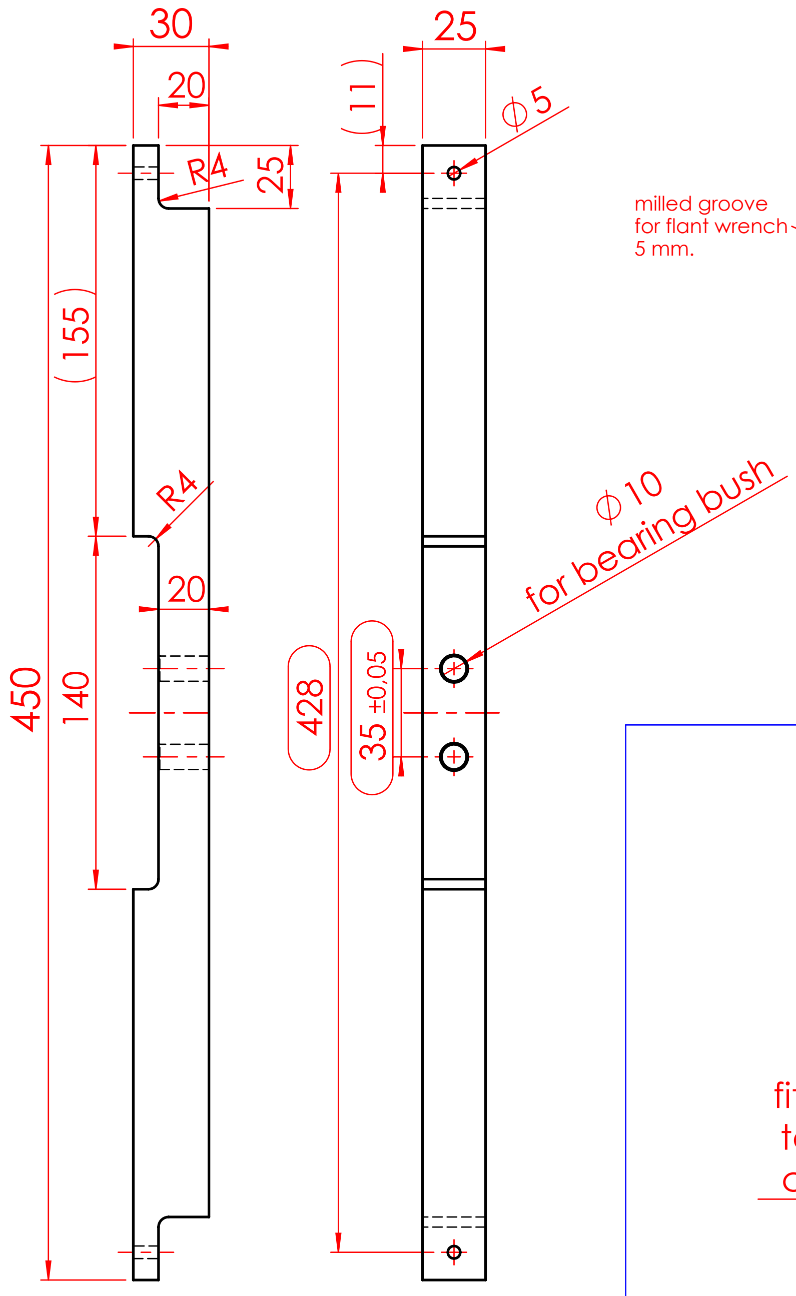

Carrier, AlMgSi 0,5
(aluminium alloy)
black anodised, 1x

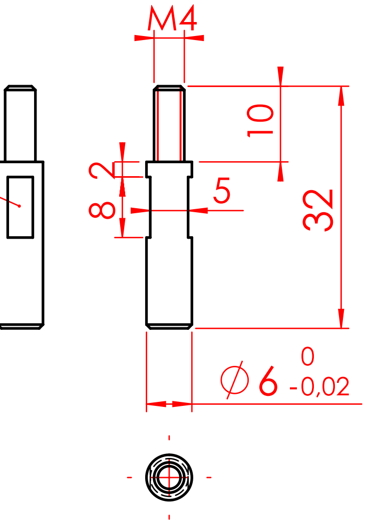

Bolt, stainless steel, 2x

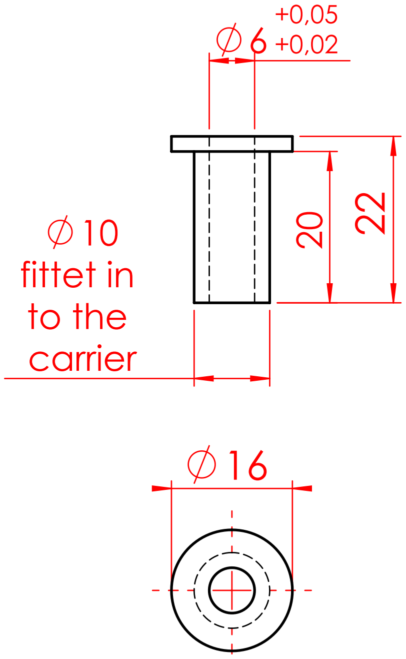

bearing bush, POM (Delrin*), 2x

(* Delrin is a brand name of DuPont)

Toleranz: DIN 7168-m	Oberfläche:	Gratfrei ohne scharfe Kanten	EMBL Heidelberg European Molecular Biology Laboratory		
		Menge: 1 unit			
	Name	Datum	Zeichen	Projekt: Instrument for photo- & chronobiology	Konto:
gez.:		2014			
gepr.:			Werkstoff:	Für: Marcus P.S. Dekens	
freigeg.				Zeichnung: Thermostat holder	A4
			Gewicht:	Maßstab 1:5	Blatt 1 von 1
