# Supplementary material for: Instrument design and protocol for the study of light controlled processes in aquatic organisms, and its application to examine the effect of infrared light on zebrafish
Source: PLoS One. 2017 Feb 17;12(2):e0172038. doi: 10.1371/journal.pone.0172038 (PMC5315407; doi:10.1371/journal.pone.0172038)
Supplement: S2 Fig — (PDF) [file pone.0172038.s002.pdf]

ALUMINIUM FRAME: INFRARED INDIRECT LIGHT BOX (SCALE 1:1)

100 mm (H) x 358 mm (L) x 358 mm (W)

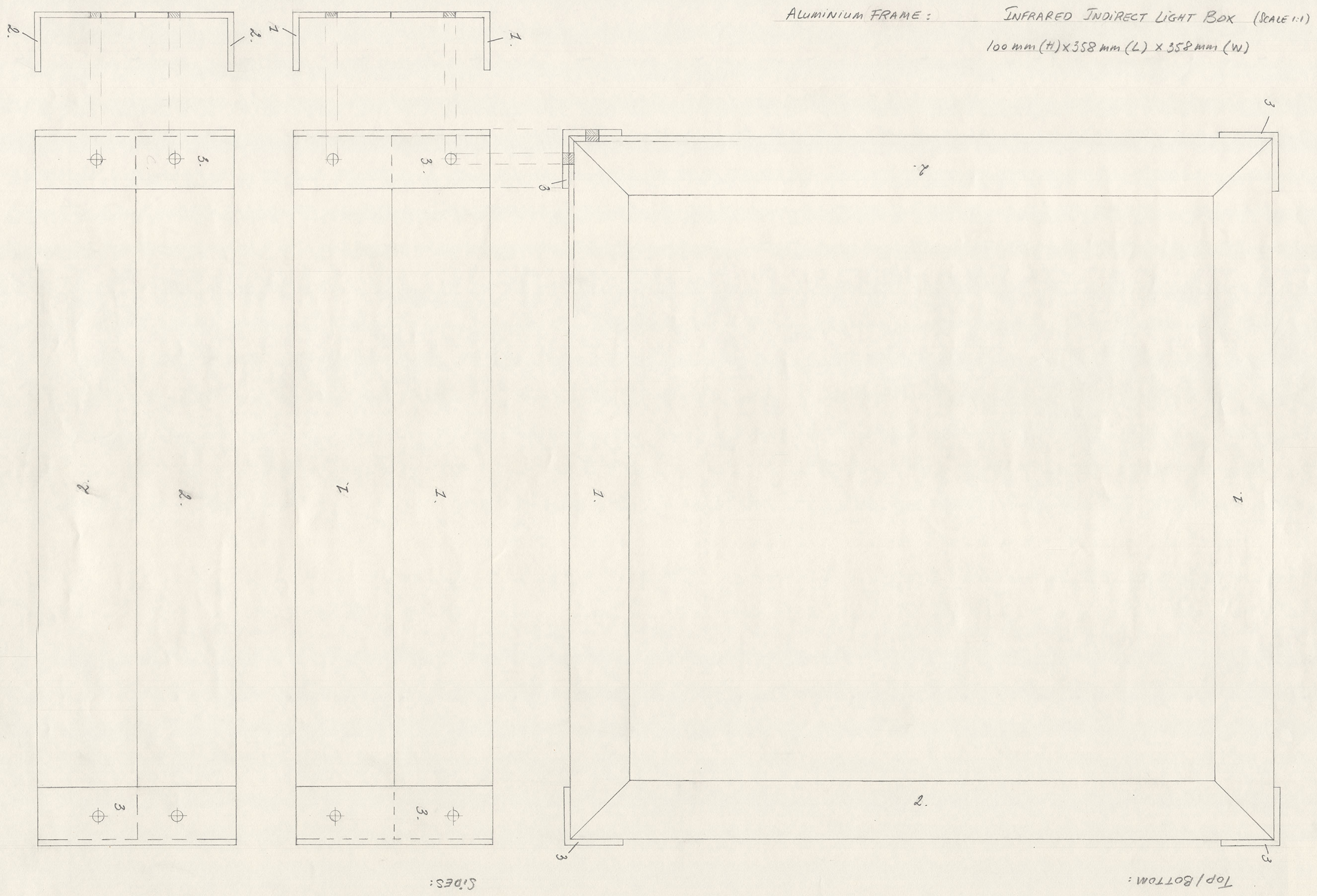

SECTION THROUGH INFRARED INDIRECT LIGHT BOX:

SCALE 1:1

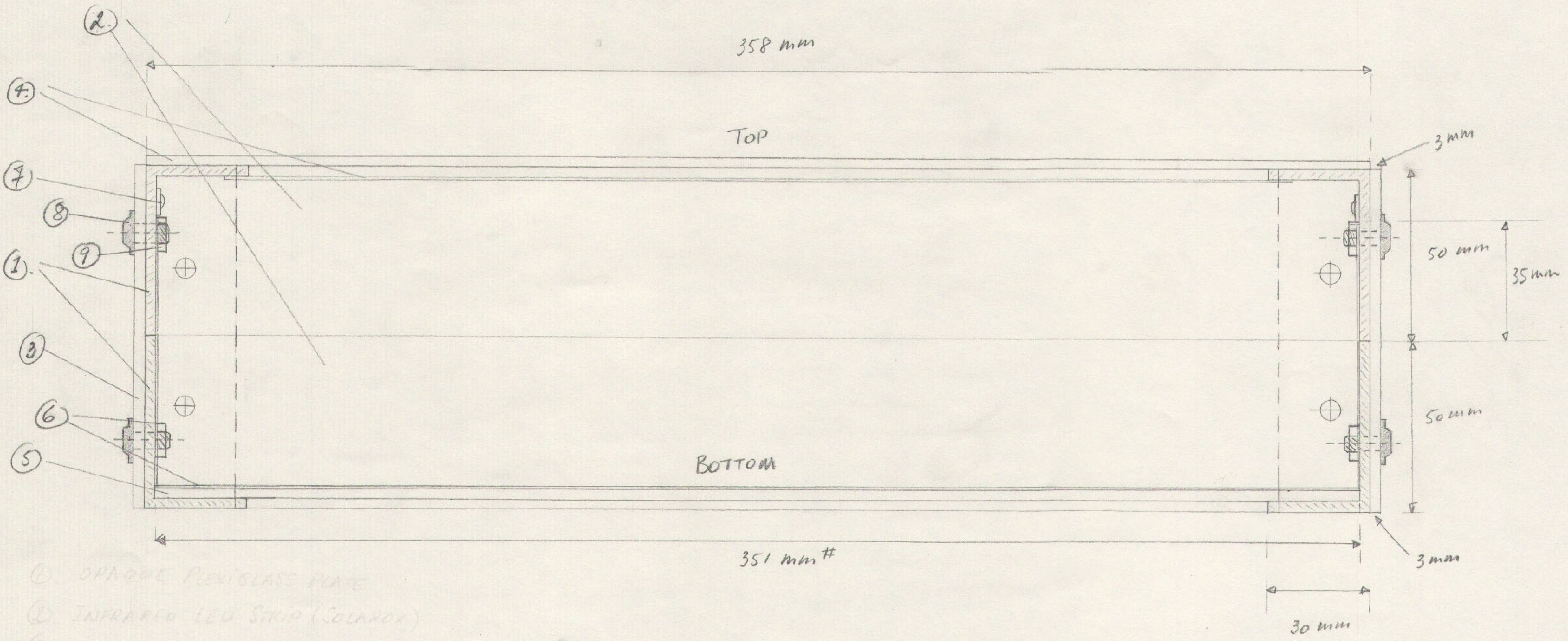

(1) OPAQUE PLEXIGLASS PLATE
(2) INFRARED LED STRIP (SOLAROX)
(3) ALUMINIUM L PROFILES
(4) PROWHITE PROJECTOR SCREEN
(5) PLEXIGLASS PLATE#

ALUMINIUM L-PROFILES (SIDES):

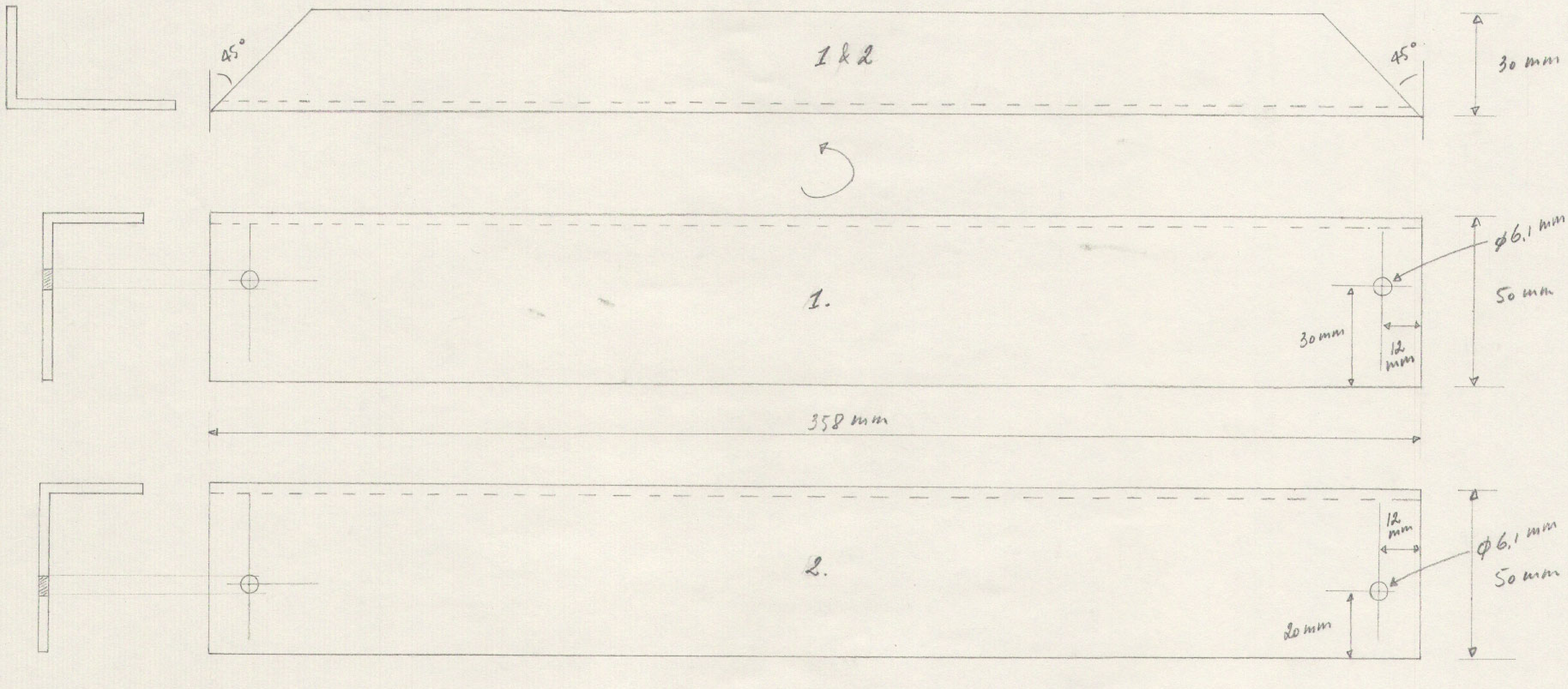

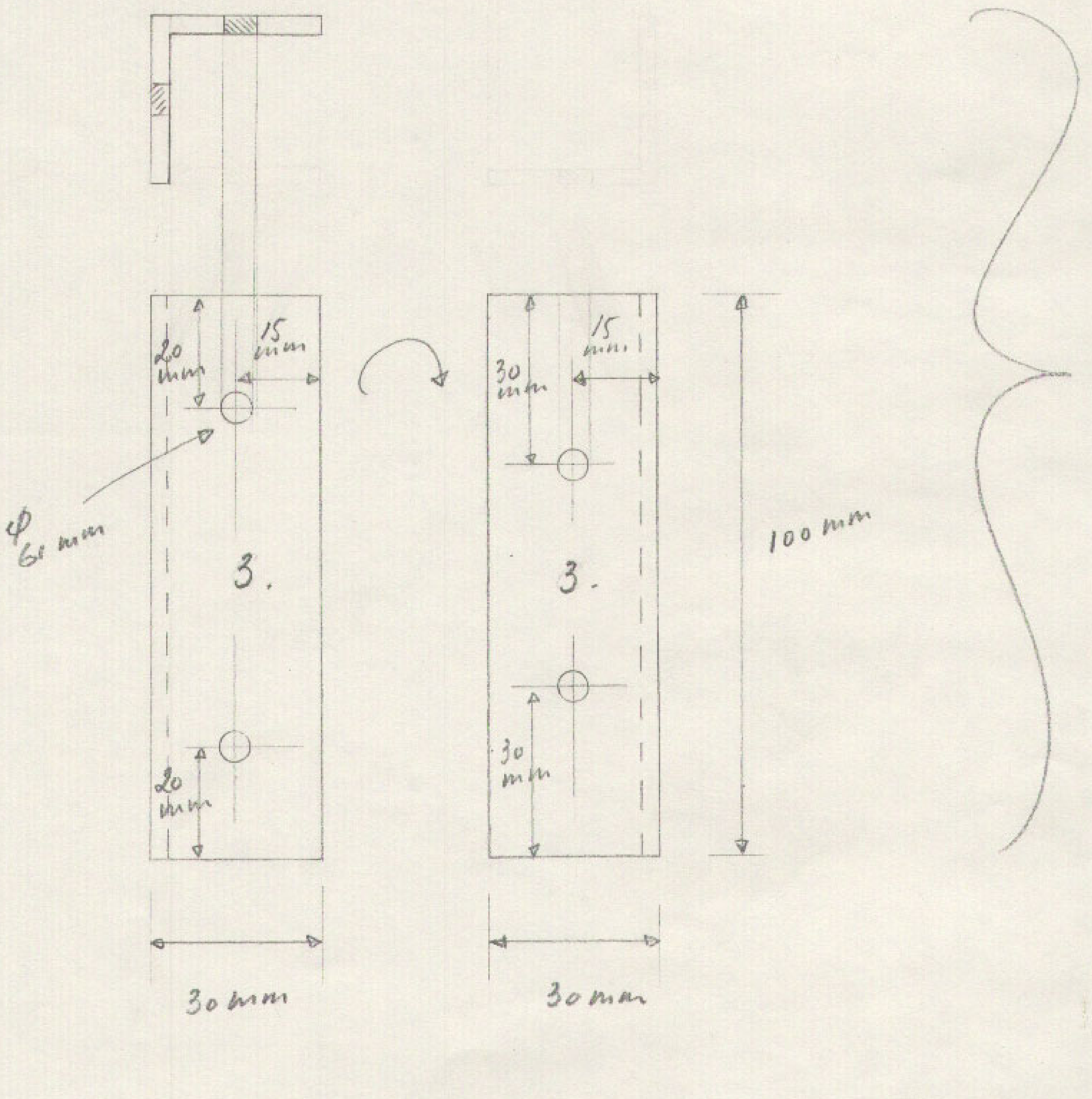

ALUMINIUM CORNER PROFILES

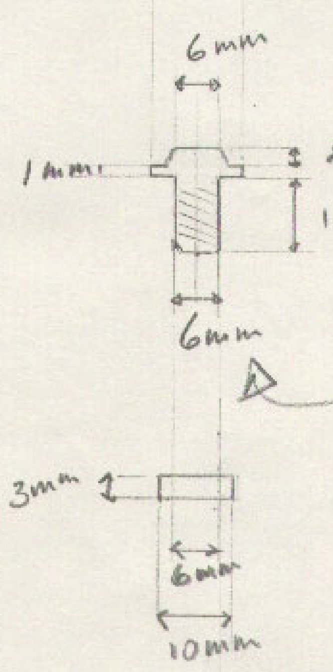

1. 4 x ALU L-PROFILE A (UNEQUAL) 358 x 30 x 50 mm
2. 4 x ALU L-PROFILE B (UNEQUAL) 358 x 30 x 50 mm
3. 4 x ALU L-PROFILE C (EQUAL) 100 x 30 x 30 mm
4. 1 X OPAQUE PLEXIGLAS TOP PLATE 3 mm & 0.7 mm (358 x 358 x 3 mm)
5. 1 X PLEXIGLAS BOTTOM PLATE# (351 x 351 x 3 mm)
6. 1 X PROWHITE RIGID PROJECTOR SCREEN
7. 1 X SOLAROX IR (840 nm) LED STRIP (140 cm)
8. 16 x HEXAGONAL SOCKET FLANGE BUTTON HEAD SCREWS → M6 10 mm A2 STEAL
9. 16 x LOW PROFILE T-NUT (ALU) M6

TEMPERATURE CONTROLLED PLATE FOR TRACKING:

BOTTOM PLATE:

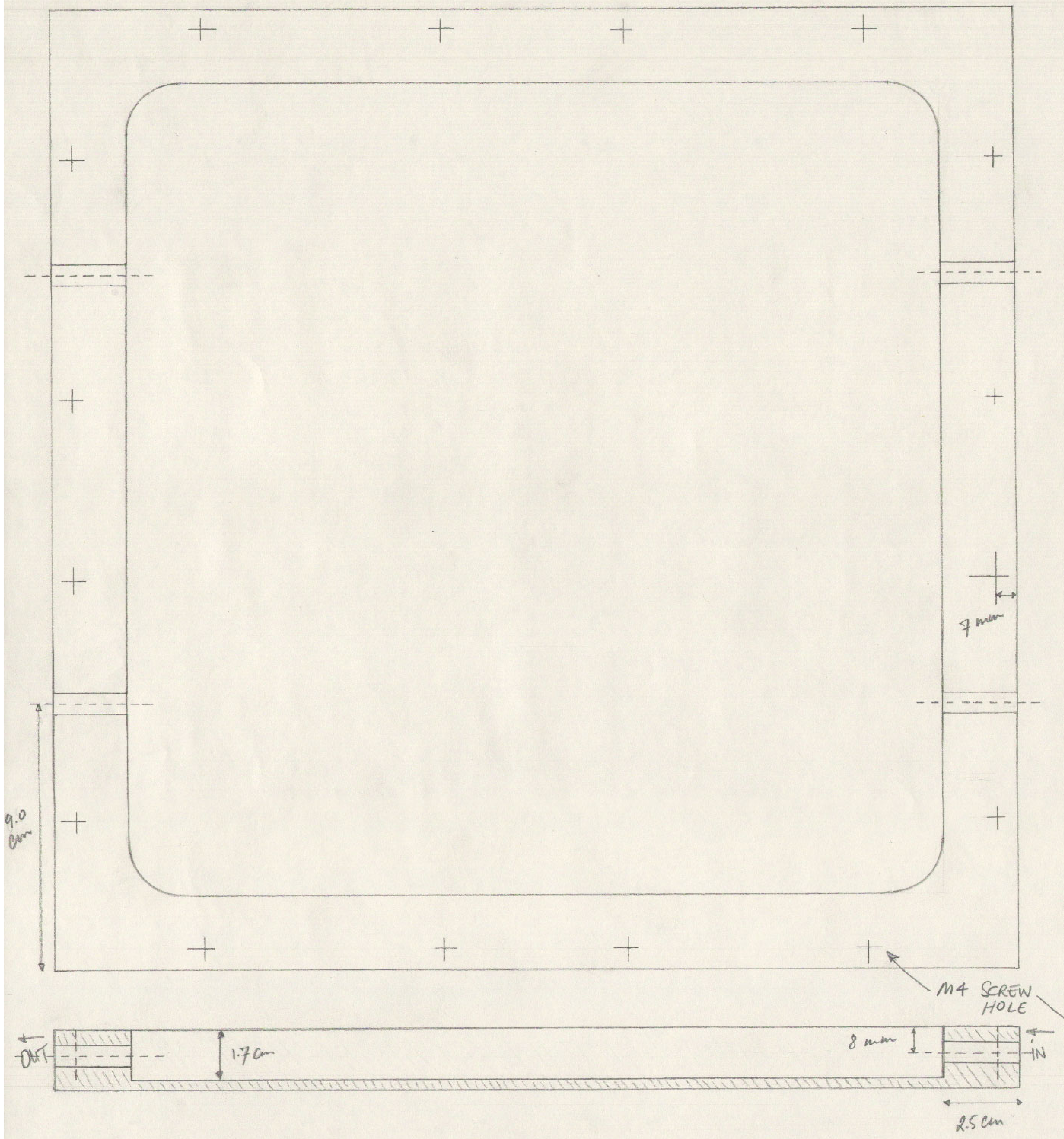

TOP PLATE:

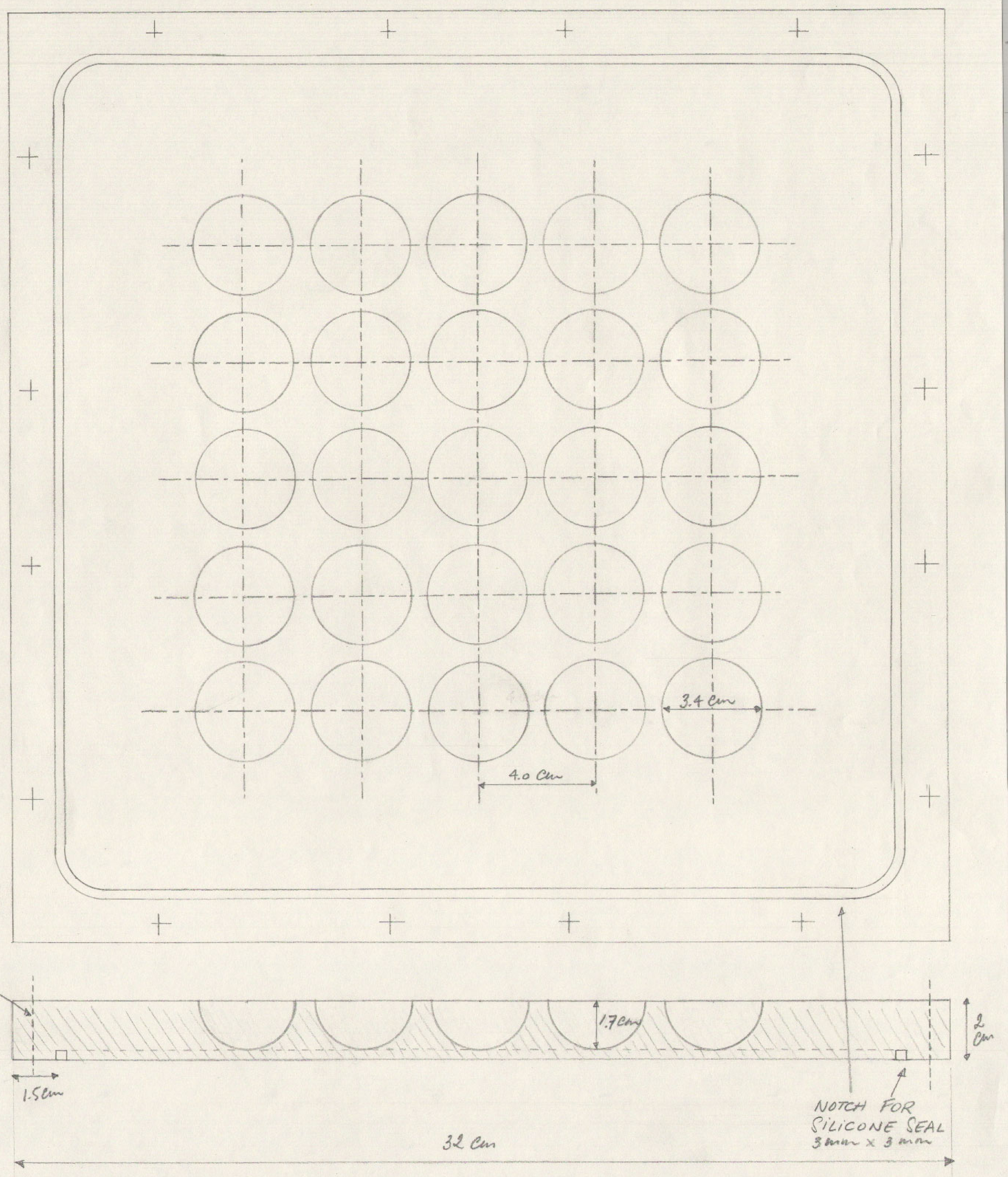

PLATE: 32 x 32 cm TRANSPARENT ACRYLIC MILLED AND SANDED.

SEAL: MATERIAL SILICONE 3mm Ø
